# Activation of class 1 integron integrase is promoted in the intestinal environment

**DOI:** 10.1371/journal.pgen.1010177

**Published:** 2022-04-28

**Authors:** Murielle Baltazar, Nadège Bourgeois-Nicolaos, Macarena Larroudé, William Couet, Solange Uwajeneza, Florence Doucet-Populaire, Marie-Cécile Ploy, Sandra Da Re

**Affiliations:** 1 INSERM, RESINFIT, U1092, Limoges, France; 2 Université Limoges, RESINFIT, U1092, Limoges, France; 3 CHU Limoges, Laboratoire de bactériologie-virologie-hygiène, Limoges, France; 4 Institut Micalis, UMR1319, Université Paris-Saclay, INRAe, AgroParisTech, Bactéries Pathogènes et Santé, Chatenay-Malabry, France; 5 AP-HP, Université Paris Saclay, Hôpital Antoine Béclère, Service de Bactériologie-Hygiène, Clamart, France; 6 Université de Poitiers, CHU Poitiers, INSERM U1070, Poitiers, France; Swiss Federal Institute of Technology Lausanne (EPFL), SWITZERLAND

## Abstract

Class 1 integrons are widespread genetic elements playing a major role in the dissemination of antibiotic resistance. They allow bacteria to capture, express and exchange antibiotic resistance genes embedded within gene cassettes. Acquisition of gene cassettes is catalysed by the class 1 integron integrase, a site-specific recombinase playing a key role in the integron system. In *in vitro* planktonic culture, expression of *intI1* is controlled by the SOS response, a regulatory network which mediates the repair of DNA damage caused by a wide range of bacterial stress, including antibiotics. However, *in vitro* experimental conditions are far from the real lifestyle of bacteria in natural environments such as the intestinal tract which is known to be a reservoir of integrons. In this study, we developed an *in vivo* model of intestinal colonization in gnotobiotic mice and used a recombination assay and quantitative real-time PCR, to investigate the induction of the SOS response and expression and activity of the class 1 integron integrase, IntI1. We found that the basal activity of IntI1 was higher *in vivo* than *in vitro*. In addition, we demonstrated that administration of a subinhibitory concentration of ciprofloxacin rapidly induced both the SOS response and *intI1* expression that was correlated with an increase of the activity of IntI1. Our findings show that the gut is an environment in which the class 1 integron integrase is induced and active, and they highlight the potential role of integrons in the acquisition and/or expression of resistance genes in the gut, particularly during antibiotic therapy.

## Author summary

Class 1 integrons are genetic systems allowing bacteria to acquire antibiotic resistance genes through the recombination activity of the IntI1 integrase. These genetic platforms are involved in the spread of antibiotic resistance among bacteria. So far, most of the studies for understanding the mechanistic of integrons have been performed *in vitro*, experimental conditions which are far from the lifestyle of bacteria in natural environments such as the gut. We developed a new *in vivo* model using gnotobiotic mice and we showed that in the gut, the basal activity of IntI1 is significantly higher than in *in vitro* conditions. In addition, we showed that a subinhibitory concentration of ciprofloxacin rapidly triggers the SOS response, leading to increased activity of IntI1 in the mouse gut. Our results demonstrate that the intestinal environment promotes the acquisition/expression of antibiotic resistance genes through the integron system and that this phenomenon can be enhanced by antibiotic therapy.

## Introduction

Overuse and misuse of antibiotics have led to the emergence of multidrug-resistant bacteria, causing a major threat worldwide [1,2]. Both pathogenic and commensal microbial species can become multidrug-resistant, and reservoirs of antibiotic resistance (AR) genes are found in various ecosystems, including the animal and human gut, soil and water [3–6]. The human gut hosts a dense microbiota that constitutes a favourable environment for the exchange of genetic material, such as AR genes, through horizontal transfer between commensal bacteria and/or incoming pathogens, and between resistant and susceptible bacteria [6–8]. Intestinal commensals shape a reservoir of AR genes known as “the gut resistome" [6,9,10] and transfer of resistance genes between bacteria has been demonstrated in the gut of gnotobiotic mice [11–14], as well as in the human gut [15].

Integrons are genetic systems that allow bacteria to capture, stockpile, express, and exchange AR genes embedded within gene cassettes. They are non-mobile *per se*, but are usually associated with mobile genetic elements such as transposons and plasmids, and thus participate actively in the dissemination of AR genes, mainly among Gram-negative bacteria [16]. Integrons are composed of an *intI* gene, which encodes a site-specific recombinase (IntI), a specific recombination site (*attI*) and a functional promoter (Pc) that allows the expression of gene cassettes, which are usually promoterless [17]. More than 130 gene cassettes have been identified, conferring resistance to almost all existing classes of antibiotics [16]. Three main classes of integrons have been associated to clinical setting, the class 1 being the predominant one [18–20]. The integrase is the key element in the integron system, catalysing both insertion and excision of gene cassettes [21,22]. The mechanisms driving its recombination activity have been extensively studied *in vitro* [23]. We have previously shown that, in *in vitro* planktonic culture, expression of the class 1 integron integrase gene, *intI1*, is regulated by the bacterial SOS response [24]. The SOS response is a global regulatory network controlled by the transcriptional repressor LexA and induced by the activator RecA when it is bound to single-strand DNA produced by stress [25]. Widely used antibiotics (trimethoprim, fluoroquinolones, β−lactams, aminoglycosides) and horizontal gene transfer (conjugation and transformation) are known to induce the SOS response *in vitro* [26–31].

Epidemiological studies have reported the presence of integrons in the healthy animal and human gut, showing that the gut is a natural reservoir of integrons [32–34]. However, to the best of our knowledge, there are no previous report on the regulation of the integron integrase and the dynamics of gene cassettes acquisition and exchange *in vivo* in the gut. In this study, we developed a model of intestinal colonization with *Escherichia coli* in gnotobiotic mice to study the expression and activity of the class 1 integron integrase and the induction of SOS regulon genes in the gut, in the absence and presence of antibiotic. Using an *in vivo* recombination activity assay and quantitative real-time PCR (qRT-PCR) from mice faeces, we provide the experimental evidence that in the mouse gut, (i) the basal activity of IntI1 is higher than in *in vitro* planktonic culture, indicating that the gut is a stressful environment able to promote antibiotic resistance gene acquisition via integrons, and (ii) administration of a subinhibitory concentration of ciprofloxacin induces both the SOS response and expression of *intI1* that is correlated with an increase of the integrase activity.

## Results and discussion

### Colonization of the mouse gut and plasmid stability

Three groups of germ-free mice were inoculated with *E*. *coli* strain MG1656 carrying two plasmids, p6851 and a pZE1-derivative, both necessary to follow the recombination activity of the class 1 integron integrase (see further and S1 Fig for more details). p6851 plasmid carried the cassette recombination reporter (Table 1 and S1 Fig). For the expression of the IntI1 integrase, we used pZE1-derivatives: pZE1intI1 allowed the expression of *intI1* from the LexA-regulated wild-type integrase promoter P*intI1*, pZE1intI1* allowed the expression of *intI1* from the promoter P*intI1* carrying mutations in the LexA binding site which led to a derepressed expression of the integrase and pZE1 was a control plasmid that did not carry the *intI1* gene, (Table 1 and S1 Fig). The strains are hereafter referred to as MG/intI1, MG/intI1* and MG/pZE1 (control strain) respectively.

**Table 1:** Bacterial strains and plasmids used in this study.

Strains and plasmids	Relevant genotype or description	Reference
*Escherichia coli strains*		
MG1656	*ΔlacMluI* derivative of *Escherichia coli* MG1655 (K-12F^–^ λ^–^ *ilvG*^*−*^*rfb-50 rph-1*)	[35]
MG/pZE1	MG1656 carrying p6851 and pZE1 plasmids	This work.
MG/intI1	MG1656 carrying p6851 and pZE1intI1 plasmids	This work.
MG/intI1*	MG1656 carrying p6851 and pZE1intI1* plasmids	This work.
MG1656*λatt*::*gfp*	MG1656 containing the *gfpmut3* gene at the *λatt* site; expression of *gfp*_*mut3*_ from P*λ* promoter. Green fluorescent bacteria.	[36]
*Plasmids*		
p6851	Cassette recombination reporter (pSU38::aac(6’)-Ib*::*attC*_*aadA7*_*-cat(T4)-attC*_*VCR2*_); Cm^R^, Km^R^	[24]
Recombined p6851	Plasmid resulting from deletion of the resistance gene cassette *cat(T4)-attC*_*VCR2*_ in native p6851; expression of the resistance gene cassette aac(6’)-Ib* from *PlacZ* promoter; Tobra^R^, Km^R^	This work.
pZE1	Promoterless derivative of pZE12 plasmid, carrying a multiple cloning site (mcs1); Amp^R^	[24]
pZE1intI1	*attI* site + *intI1* gene from In40 class 1 integron cloned into pZE1 plasmid; this *intI1* gene encodes the most active variant of the integrase, IntI1_R32_H39_; Amp^R^	[24,37]
pZE1intI1*	pZE1intI1 plasmid carrying the LexAmut2 mutation in the LexA binding site of the class 1 integron integrase promoter P*intI1*, leading to constitutive expression of the *intI1* gene; Amp^R^	[24]

Cm^R^: confers resistance to chloramphenicol; Km^R^: confers resistance to kanamycin; Tobra^R^: confers resistance to tobramycin; Amp^R^: confers resistance to ampicillin

**Fig 1:**
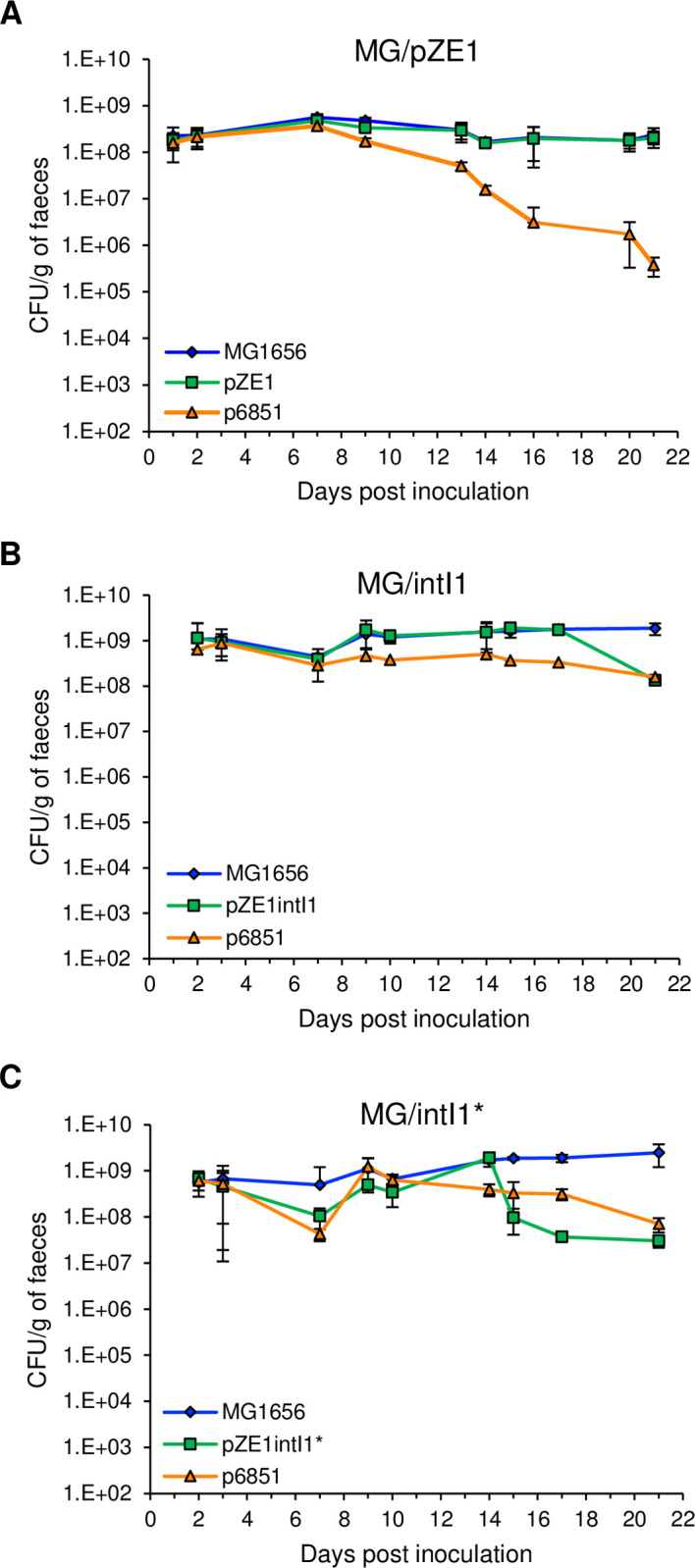
Bacterial colonization and plasmid stability in the mouse gut. On day 0, three groups of germ-free mice were inoculated with 10^8^ CFU of MG/pZE1 control strain (carrying p6851 and pZE1 that did not carry the *intI1* gene, n = 4) **(A)**, MG/intI1 strain (carrying p6851 and pZE1intI1 allowing the expression of *intI1* SOS-regulated, n = 4) **(B)** or MG/intI1* strain (carrying p6851 and pZE1intI1* allowing the constitutive expression of *intI1*, n = 3) **(C)**. Bacterial colonization in the mouse gut was monitored by counting the CFU/g of faeces on non-selective medium (MG1656). Plasmid stability was evaluated by counting the CFU/g of faeces on selective media supplemented with kanamycin (p6851) or ampicillin (pZE1, pZE1intI1 and pZE1intI1*). Symbols represent the average of the CFU/g of faeces per day and error bars indicate the SD.

First, we verified the capability of *E*. *coli* strain MG1656 to colonize the mouse gut, as well as the stability of the plasmids throughout the duration of the experiments. Colony-forming units (CFU) counting from mice faeces on non-selective medium, showed that MG/pZE1, MG/intI1 and MG/intI1* readily colonized the mouse gut and established at high levels throughout the 21 days of experiment, at an average population level of 2.9x10^8^ ± 1.4x10^8^, 1.3x10^9^ ± 4.4x10^8^ and 1.3x10^9^ ± 7.3x10^8^ CFU/g of faeces respectively (Fig 1). The stability of plasmids p6851 and pZE1-derivatives in bacteria, was monitored by CFU counting on selective media supplemented with kanamycin or ampicillin respectively. Plasmid instability was defined by a recovery of bacteria on selective media significantly lower (p < 0.05) than the recovery of the total bacterial population on non-selective medium. Control plasmid pZE1 was stable throughout the experiment, pZE1intI1 and pZE1intI1* were stable in bacteria up to day 17 and day 14 post inoculation respectively (Fig 1). The stability of p6851 plasmid was variable from up to day 10 to up to day 17 post inoculation according to the experiments. The observed differences in the stability of the plasmids expressing the integrase, pZE1intI1 and pZE1intI1* suggests that integrase overexpression might be deleterious and that the fitness cost might be too high for bacteria, leading to the loss of the plasmid in the bacterial population. Previous studies suggested that an unregulated integrase activity was disadvantageous for bacteria [38,39]. Recently we showed that the fitness cost of the class 1 integrase was correlated to its catabolic activity and that the SOS response prevented the expression of this costly integrase in *E*. *coli* [36]. Similarly, Starikova *et al*. observed that the expression of an active integrase reduced host fitness in *Acinetobacter baylyi* in the absence of regulation of the *intI1* gene [40].

### Class 1 integron integrase is active in the gut

**Fig 2:**
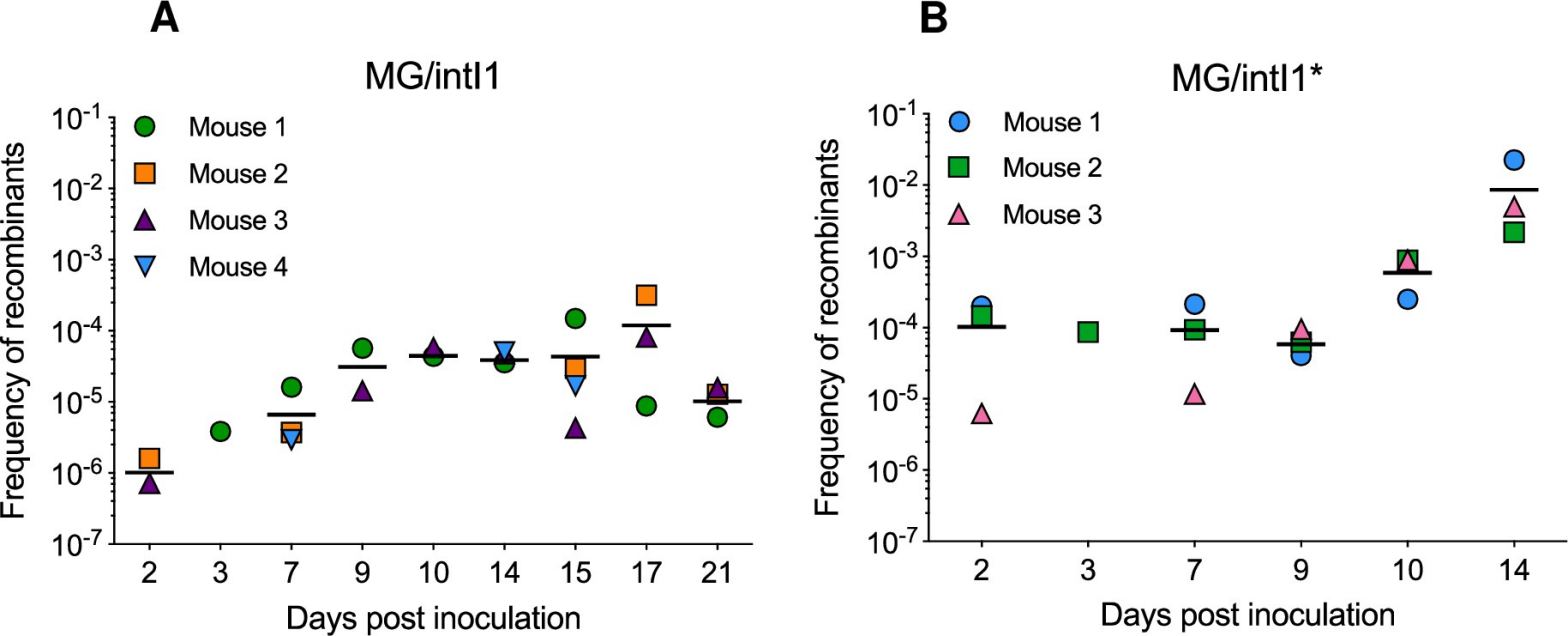
Recombination activity of IntI1 integrase in the mouse gut. On day 0, two groups of germ-free mice were inoculated with 10^8^ CFU of MG/intI1 (carrying p6851 and pZE1intI1 allowing the expression of *intI1* SOS-regulated, n = 4) **(A)** or MG/intI1* (carrying p6851 and pZE1intI1* allowing the constitutive expression of *intI1*, n = 3) **(B)**. Recombination activity of IntI1, reflected by the frequency of recombinants (FR), was estimated by determining the frequency of emergence of tobramycin-resistant recombinants, as a result of specific recombination between *attC* sites located on a synthetic array of two cassettes (*attC*_*aadA7*_-*cat(T4)*-*attC*_*VCR2*_-aac(6’)-Ib*) carried on plasmid p6851. Each symbol represents the FR in the gut calculated from a single mouse exhibiting recombinants. For each sampling day, the average FR is shown as a black horizontal line.

In order to estimate the activity of the IntI1 integrase in the mouse gut, we assessed the capability of IntI1 to catalyse specific recombination between *attC* sites located on a synthetic array of two cassettes (*attC*_*aadA7*_-*cat(T4)*-*attC*_*VCR2*_-aac(6’)-Ib*) carried by plasmid p6851, resulting in the deletion of the chloramphenicol resistance gene cassette *cat(T4)-attC*_*VCR2*_, and the expression of the tobramycin resistance gene cassette aac(6’)-Ib* (S1 Fig). The recombination activity of the integrase was thus determined by monitoring the emergence of tobramycin-resistant (Tobra^R^) bacteria, hereafter referred to as recombinants, as a function of the total bacterial population carrying the two plasmids and was reported as the frequency of recombinants (FR). As expected, when no integrase was expressed (MG/pZE1 strain), no recombinants were recovered. In mice colonized with MG/intI1, recombinants were detected on day 2 post inoculation (S2A Fig), reflecting that the IntI1 integrase carried out recombination events. The average FR increased gradually from day 2 (1.2x10^-6^ ± 6.3x10^-7^) to day 9 (3.6x10^-5^ ± 3.0x10^-5^), then it stabilized until day 17 post inoculation (Fig 2A). The gradual increase of the FR corresponded to an increase of the number of recombinants (tobramycin-resistant) and their stable establishment in the mouse gut (S2A Fig). In addition, we observed a variability of the FR from one mouse to another, as well as a temporal variability in the detection of recombinants, where the number of mice exhibiting recombinants varied from one to four mice depending on the sampling day. Indeed, before day 14 post inoculation, 50% of mice (10/20) were colonized, then 81% of mice (13/16) were colonized until the end of the experiment (Fig 2A). To assess whether the variation in recombinants detection was not due to differences in fitness cost between the native non-recombined p6851 and the recombined plasmid, we performed *in vitro* pairwise competition assays between two MG1656 strains carrying either the native non-recombined p6851 or the recombined plasmid. As shown in S3 Fig, both plasmids had similar fitness cost, with no strain being fitter than the other. During the first ten days, the population of recombinants did not seem to only maintain itself at a constant level but rather resulted from a combination of the maintenance of the recombinants and the appearance of new recombination events over time. PCR and sequencing analysis confirmed that recombinants resulted mainly from recombination between the two *attC* sites of plasmid p6851. Indeed, most of the recombinants were susceptible to chloramphenicol (Cm^S^). These results contrast with previous data obtained in planktonic culture, where recombination events mediated by IntI1, mainly generated plasmid co-integrates, i.e. recombination between *attC* sites of two copies of the same plasmid and/or duplicated gene cassettes [22,41,42]. Our results provide the first experimental evidence that the IntI1 integrase is active in the gut and that it efficiently ensures recombination events.

We also estimated the FR in mice colonized with MG/intI1* (constitutive expression of *intI1*), considering only the period during which both plasmids were stable (from day 2 to day 14 post inoculation) (Fig 1C). Contrary to the experiment carried out with MG/intI1, when colonized with MG/intI1*, all mice were colonized with recombinants since day 2 post inoculation (Fig 2B), that reflects that IntI1 catalysed recombination events and that recombinants established quickly in the mouse gut. On day 2, the average number of recombinants was 1.5 log_10_ higher than the one observed in the gut of mice colonized with MG/intI1 (S2B Fig), indicating that the disruption of the LexA binding site in P*intI1* had the same effect *in vivo* as in planktonic cultures. This observation suggests that in the gut P*intI1* could also be under the control of LexA. The FR was stable over the nine first days (9.4x10^-5^ ± 2.2x10^-5^), and then increased up to 9.9x10^-3^ ± 1.1x10^-2^ on day 14 post inoculation (Fig 2B). Although the integrase was constitutively expressed in MG/intI1*, the population of recombinants did not increase over the nine first days but reached 2.3x10^4^ ± 1.9x10^3^ CFU/g of faeces (on day 9 post inoculation), which was 4.0 log_10_ lower than the total bacterial population (S2B Fig). Then, there was a further increase of the FR from day 10 to day 14 post inoculation (Fig 2B) that correlated with an increase of the number of recombinants (S2B Fig), suggesting that some recombinants started to proliferate in the gut. This latter bacterial population still carrying both plasmids, was predominant towards the end of the experiment: on day 17 post inoculation, the total bacterial population and the population of recombinants were respectively 1.3x10^7^ ± 7.6x10^6^ and 2.6x10^6^ ± 3.5x10^6^ CFU/g of faeces (p > 0.05) (S2B Fig).

**Fig 3:**
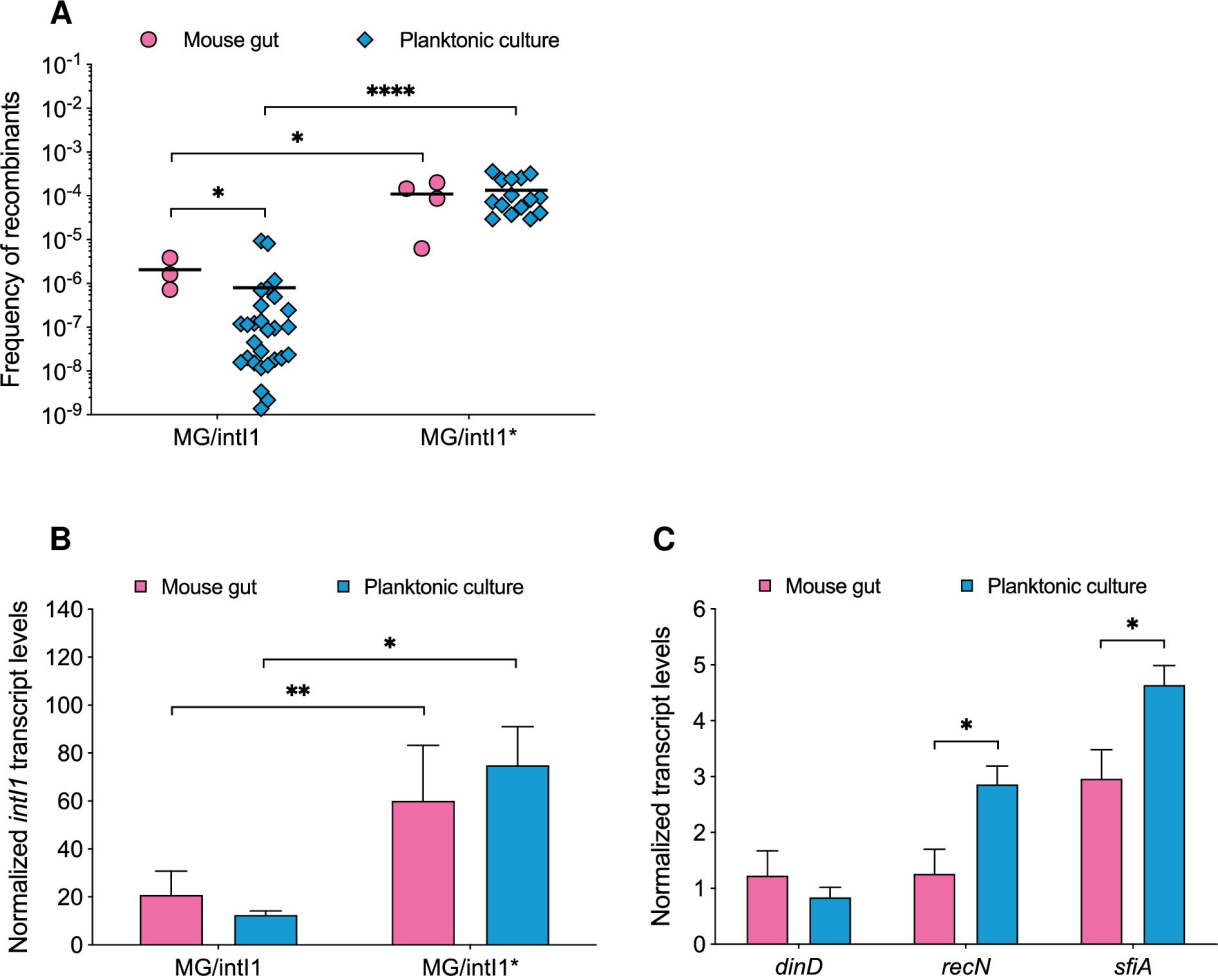
Comparisons of recombination activity of IntI1 and expression of *intI1* and SOS regulon genes, *in vivo versus in vitro*. **(A)** Recombination activity of IntI1, reflected by the frequency of recombinants (FR), was estimated by determining the frequency of emergence of tobramycin-resistant recombinants, as a result of specific recombination between *attC* sites located on a synthetic array of two cassettes (*attC*_*aadA7*_-*cat(T4)*-*attC*_*VCR2*_-aac(6’)-Ib*) carried on plasmid p6851. The FR was calculated in MG/intI1 (carrying p6851 and pZE1intI1 allowing the expression of *intI1* SOS-regulated) and MG/intI1* (carrying p6851 and pZE1intI1* allowing the constitutive expression of *intI1*), in the mouse gut and in 24-h-old-planktonic cultures. Each symbol represents the FR calculated from a single mouse exhibiting recombinants on days 2 and 3 post inoculation (n = 3 for MG/intI1 and n = 4 for MG/intI1*) and from planktonic cultures (n = 28 for MG/intI1 and n = 15 for MG/intI1*). For each condition, the average FR is shown as a black horizontal line. Transcript levels of **(B)** the *intI1* gene in MG/intI1 and MG/intI1* and **(C)** the SOS regulon genes *dinD*, *recN* and *sfiA*, in MG/intI1 in the mouse gut and in 24-h-old-planktonic cultures are represented. Transcript levels were normalized to the housekeeping gene *dxs*. Error bars indicate the SD. Differences were determined using the Mann-Whitney *U* test. *p < 0.05, **p < 0.01 and ****p < 0.0001.

Interestingly, the average FR determined in MG/intI1, once stabilized (between days 10 to 17 post inoculation), was similar to the average FR determined in MG/intI1* between days 2 to 9 post inoculation (7.0x10^-5^ ± 8.7x10^-5^ *versus* 9.6x10^-5^ ± 7.2x10^-5^ respectively; p > 0.05) (Fig 2). This observation suggested that in both groups of mice, a similar equilibrium of the population of recombinants was reached and resulted from both excision events and the establishment of recombinants in the gut. In addition, we compared the activity of IntI1 *in vivo* (considering days 2 and 3 post inoculation) and in 24-h-old-planktonic cultures. In MG/intI1, the average FR was 2.6-fold higher in the mouse gut than in 24-h-old-planktonic cultures (2.1x10^-6^ ± 1.6x10^-6^ and 8.0x10^-7^ ± 2.3x10^-6^ respectively, p < 0.05) (Fig 3A), whereas it was similar in both experimental conditions in MG/intI1* (1.3x10^-4^ ± 1.1x10^-4^ in 24-h-old-planktonic cultures *versus* 1.1x10^-4^ ± 8.3x10^-5^ in the mouse gut) (Fig 3A). Moreover, the average FR was significantly higher in MG/intI1* than in MG/intI1 in both experimental conditions (p < 0.05 and p < 0.0001 for the mouse gut and 24-h-old-planktonic cultures respectively) (Fig 3A). Altogether, our results indicate that the lifestyle of bacteria in the gut promotes the induction of the expression of *intI1* and thus the activity of the IntI1 integrase.

We then quantified by qRT-PCR the transcript levels for *intI1* and three SOS regulon genes: *dinD*, *recN* and *sfiA*, in the mouse gut (considering days 2 and 3 post inoculation) and in 24-h-old-planktonic cultures. Interestingly, in MG/intI1, transcript levels of *intI1* and *dinD* genes did not differ significantly (p > 0.05) (Fig 3B and 3C), while those of *sfiA* and *recN* genes were respectively 1.6- and 2.3-fold significantly lower in the mouse gut than in 24-h-old-planktonic cultures (p < 0.05) (Fig 3C). As expected, the constitutive expression of *intI1* was correlated with a level of transcription significantly higher in MG/intI1* than in MG/intI1, both in the mouse gut and in 24-h-old-planktonic cultures (respectively 2.9-fold and 6.0-fold; p < 0.01 and p = 0.05 respectively) (Fig 3B). Thus, as previously described in *in vitro* planktonic culture, the regulation of class 1 integron integrase expression by the SOS response via the repressor LexA, appears to be efficient in the mouse gut. In planktonic culture, SOS genes are known for showing different expression profiles: they do not respond to the same level of stress, at the same time, and they are not expressed at the same level [43,44]. Our results confirm that in the mouse gut as observed *in vitro*, the basal expression of SOS-regulated genes varies, depending on the gene, with expression levels that are similar (*intI1*, *dinD*) whatever the condition, or lower (*sfiA*, *recN*) *in vivo* than in *in vitro* planktonic cultures (Fig 3B and 3C).

In a previous study, we showed in a biofilm model that in addition to the SOS-dependent regulation of IntI1, the stringent response exerts a biofilm-specific regulation of *intI1* expression, part of this regulation being SOS-dependent and the other one involving the protease Lon [45]. In the gut, bacteria form biofilms on the epithelial mucosa and on various particles present in the lumen [46,47]. The stringent response, which is induced upon nutrient starvation [48–50], might also participate in the upregulation of *intI1* in the intestinal tract. Biofilms are known to have a heterogeneous spatial structure with differences in nutrient availability [51], and several studies have shown that available nutrients in the gut vary in time and space [52]. It is thus more than likely that, individually, bacteria do not experience the same nutrient deprivation at the same level and/or at the same time within the gut. We can hypothesize that the induction of the *intI1* expression by the stringent response, has occurred in a minority of bacteria dispersed within the total population. As a result, the induction of *intI1* expression could not have been detected from the total bacterial population using the quantification of transcript levels; however, it could be estimated via the recombination activity assay of the integrase using antibiotic selection and counting of individual bacteria having experienced recombination events.

### Ciprofloxacin induces the SOS response and expression of IntI1 in the gut

**Fig 4:**
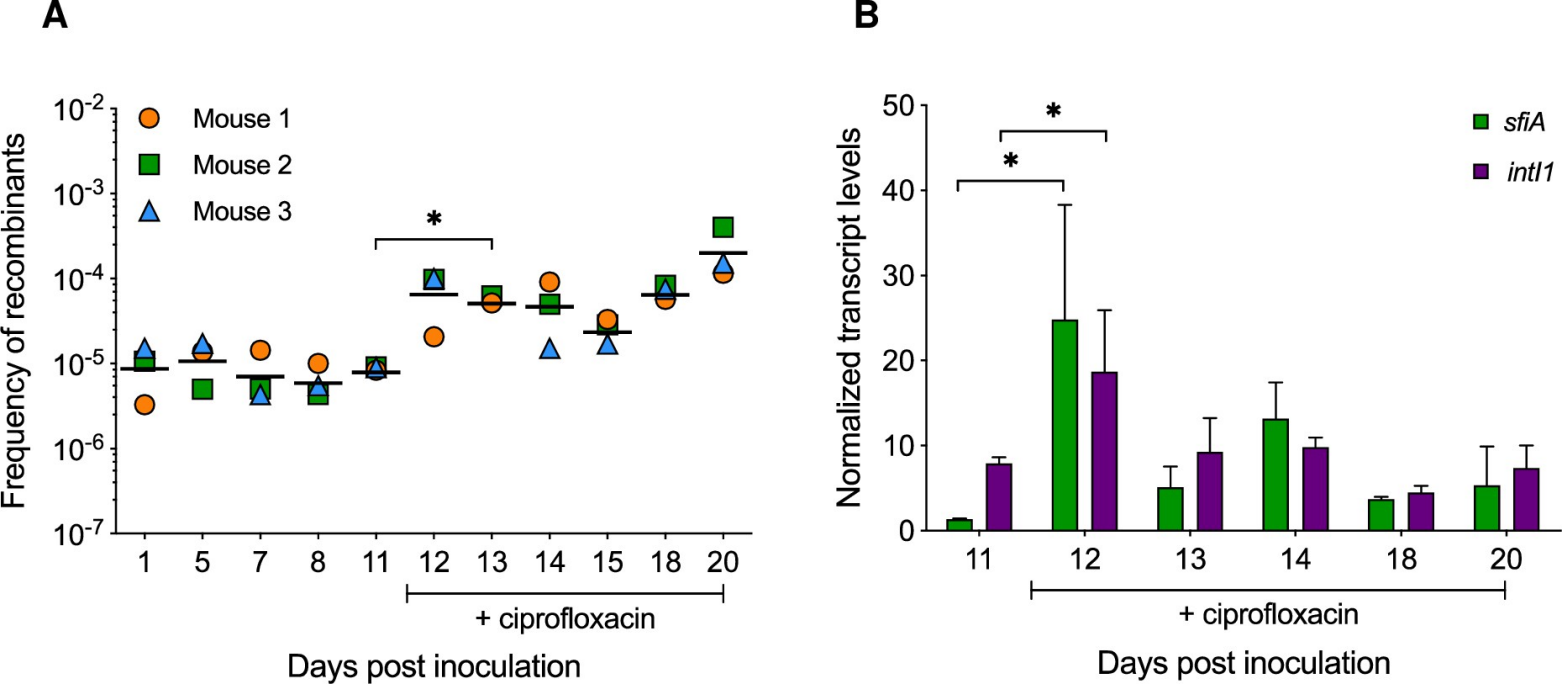
Effect of ciprofloxacin on the induction of the SOS response and the IntI1 integrase expression and activity in the mouse gut. On day 0, three germ-free mice were inoculated with 10^8^ CFU of MG/intI1 (carrying p6851 and pZE1intI1 allowing the expression of *intI1* SOS-regulated). Ciprofloxacin was added to the drinking water of mice on day 11 post inoculation, just after the fecal sampling on that day. **(A)** Recombination activity of IntI1, reflected by the frequency of recombinants (FR), was estimated by determining the frequency of emergence of tobramycin-resistant recombinants, as a result of specific recombination between *attC* sites located on a synthetic array of two cassettes (*attC*_*aadA7*_-*cat(T4)*-*attC*_*VCR2*_-aac(6’)-Ib*) carried on plasmid p6851. Each symbol represents the FR in the gut calculated from a single mouse exhibiting recombinants. For each sampling day, the average FR is shown as a black horizontal line. **(B)** Transcript levels of *sfiA* and *intI1* genes in the mouse gut are represented. Transcript levels were normalized to the housekeeping gene *dxs*. Error bars indicate the SD. Differences were determined using the Mann-Whitney *U* test. *p < 0.05.

Ciprofloxacin is known to induce the SOS response and thereby the expression of *intI1* in *E*. *coli in vitro* [24]. To evaluate the effect of ciprofloxacin on the integrase induction in the gut, we continuously exposed mice colonized with MG/intI1 to ciprofloxacin in the drinking water. Based on a previous study about the impact of ciprofloxacin in a human-flora-associated mouse model [53], we first assessed three different doses of ciprofloxacin: 0.1, 1 and 10 mg of ciprofloxacin/kg of body weight per day, which corresponded to ciprofloxacin concentrations in the drinking water of 0.4 μg/ml, 4 μg/ml and 40 μg/ml respectively (S1 Table). Ciprofloxacin doses of 1 and 10 mg/kg were lethal to bacteria (S1 Table) whereas a dose of 0.1 mg/kg led to only a slight decrease of the intestinal bacterial population that however remained high in the mouse gut (S1 Table and S4A Fig). Thus, this ciprofloxacin dose has been used to study its effect on the *in vivo* expression and activity of IntI1. Once the bacterial population was stably established in the mouse gut (S4B Fig), on day 11 post inoculation, ciprofloxacin was added to the drinking water of mice until the end of the experiment. We determined the antibiotic concentration in the mouse gut: 0.3 ± 0.2 μg/g of faeces were detected throughout the experiment, showing that ciprofloxacin concentration was stable in the gut over time (S1 Table). We found that ciprofloxacin induced a 0.9 log_10_ significant increase of the FR of IntI1, within 48h following its introduction in the drinking water (p < 0.05) (Fig 4A). Although ciprofloxacin induced a slight decrease in the total bacterial population, the population of recombinants remained stable (S4B Fig), reflecting the appearance of new recombination events and the establishment and proliferation of the recombinants in the mouse gut. In addition, we quantified the *in vivo* transcript levels of *intI1* and *sfiA* genes. The *sfiA* gene was used as a positive control of the SOS response as it is known to be strongly induced following bacterial stress [54]. As shown in Fig 4B, 24h following the antibiotic introduction (day 12 post inoculation), we observed an increase of transcript levels of *sfiA* and *intI1*, 18.5-fold and 2.4-fold respectively (p = 0.05). Then, the levels of transcription of both genes decreased gradually, returning almost to their basal expression level (Fig 4B), suggesting that bacteria have adapted to the ciprofloxacin-containing environment, restoring the LexA control that allowed them to respond to new incoming stress. These results are consistent with a previous study showing that the SOS response induced by sublethal ciprofloxacin concentrations increased the fitness of *Pseudomonas aeruginosa in vitro* [55]. The authors found that the SOS response expression in *P*. *aeruginosa* increased substantially upon exposure to ciprofloxacin but then declined rapidly by 50%, concluding that adaptation to stress decreased the expression of the SOS response pathway [55]. In our experiment, the FR remained high after ciprofloxacin exposure (Fig 4A). This might be explained by the proliferation and the maintenance of the recombinants, resulting from the induction of *intI1* expression just after the introduction of the antibiotic (Fig 4B), in addition to ongoing new recombination events, *intI1* still being expressed at its basal level. Throughout the experiment, no ciprofloxacin-resistant isolates were recovered. The minimal inhibitory concentration of ciprofloxacin for the recombinants recovered prior and following ciprofloxacin administration was unchanged (0.012 μg/ml), indicating that bacteria have adapted to the ciprofloxacin-containing environment in the gut without acquiring ciprofloxacin resistance. Thus, a ciprofloxacin dose as low as 0.1 mg/kg of body weight per day, which was more than 100-fold lower than the usual human therapeutic dose, was sufficient to induce the SOS response and stimulate the upregulation of the class 1 integron integrase in the mouse gut. In the human and animal intestinal tract, commensal bacteria can encounter subinhibitory concentrations of antibiotics [56,57], that can affect gene transcription [58], trigger the transfer of mobile genetic elements carrying AR genes [59,60], and select resistant bacteria as well as multidrug-resistance plasmids [61–65]. Herein, we showed that a subinhibitory concentration of ciprofloxacin is sufficient to trigger the SOS response in the gut, inducing the integrase expression that could thus potentially lead to antibiotic resistance gene cassette acquisition, or promote shuffling of gene cassettes within integrons, bringing a distal gene cassette closer to the Pc promoter and thereby allowing or enhancing its expression. A such event has been observed in a hospitalized patient infected by a *P*. *aeruginosa* strain carrying a class 1 integron [66]. Indeed, following treatment with ceftazidime and metronidazole (to treat a secondary infection by anaerobes), metronidazole led to the SOS induction in *P*. *aeruginosa*, which promoted the excision of a gene cassette by IntI1, thereby enabling the full expression of a downstream *bla*_OXA-28_ gene cassette encoding an extended-spectrum beta-lactamase responsible for the high resistance to ceftazidime of this clinical isolate [66].

It had been suggested that integrase expression yields some toxicity and that bacteria lacking LexA would be more prone to display a loss of integrase functionality [38]. We thus investigated whether MG/intI1 bacteria colonizing the mice gut acquired mutations inactivating/lowering the expression of the integrase by sequencing the region encompassing the P*intI1* promoter to the end of *intI1* gene from recombinant clones (resistant to tobramycin and susceptible to chloramphenicol) that were recovered at different times after the addition of ciprofloxacin to the drinking water of mice (between days 12 and 18 post inoculation; Fig 4). We found no mutation, either in the promoter or in the *intI1* gene. This might not be so surprising since the induction of the integrase expression by the ciprofloxacin is restricted in time, with bacteria adapting to the stress (Fig 4B). Furthermore, the cost of the integron integrase is not very high in *E*. *coli* [36].

In this study, we used germ-free mice colonized with a single *E*. *coli* strain (MG/pZE1, MG/intI1 or MG/intI1*). This animal model hosting a very simple microbiota is obviously much less complex than the human gut microbiota. However, this first simple model was useful as proof of concept to study the dynamics of expression of the integron integrase and the SOS response induction *in vivo*. It allowed us to show that the gut itself, in the absence of competition or influence from other microbial strains/species, is an environment that promotes (i) the integron integrase activation, potentially allowing bacteria to acquire antibiotic resistance genes from the gut resistome, (ii) the quick induction of the SOS response in the presence of SOS-inducer antibiotics, enabling bacteria to cope with and adapt to exogenous stress such as low antibiotic concentrations that they could potentially encounter in the gut [56,57]. Next step would be to develop a human microbiota-associated mouse model, to investigate to which extend the human intestinal microbiota could affect the dissemination of antibiotic resistance genes via integrons in the gut resistome.

In addition to extend our understanding of the regulation and the dynamics of expression of the integron integrase in the intestinal tract, our results also raise again questions as to the impact of the use of antibiotics known to be SOS-inducers, on bacterial genetic adaptation and on the acquisition of genetic determinants of drug resistance within the gut. In agreement with previous studies [67,68], our findings highlight the potential of the SOS system as a good candidate for the development of new therapeutic strategies to prevent the acquisition and spread of antibiotic resistance genes among bacteria.

## Materials and methods

### Ethics statement

The protocols involving animals and their care were conducted in conformity with the institutional guidelines that are in compliance with national and international laws and policies. All efforts were made to minimize animal suffering. The protocol was approved by the Committee on the Ethics of Animal Experiments n°26 of the University of Paris-Sud (n°2012–120). Experiments were conducted according to the European Union regulations (Directive 210/63 UE) for animal experiments and complied with our institution’s guidelines for animal care and handling.

### Bacterial strains, plasmids and growth conditions

The bacterial strains and plasmids are listed in Table 1. *E*. *coli* MG1656 cells were freshly electroporated simultaneously with plasmid p6851 and a pZE1 derivative plasmid *i*.*e*. pZE1 (MG/pZE1 strain), pZE1intI1 (MG/intI1 strain) or pZE1intI1* (MG/intI1* strain) (Table 1 and S1 Fig), then plated on Lysogeny Broth (LB) agar medium supplemented with ampicillin (Amp, 100 μg/ml) and chloramphenicol (Cm, 25 μg/ml), and incubated at 37°C overnight. For inoculation of germ-free mice with MG/pZE1, MG/intI1 or MG/intI1*, four colonies were grown in brain-heart infusion (BHI) broth without antibiotics at 37°C for seven hours statically. For competition assays, bacterial strains were grown in Davis Minimal (DM) broth supplemented with 25 μg/ml glucose (DM25 medium).

### Model of intestinal colonization in gnotobiotic mice

#### Mice

C3H germ-free female mice (6–8 weeks, 20 g) obtained from Cryopreservation, Distribution, Typage et Archivage animal (CDTA) (CNRS, Orléans, France), were housed in sterile isolators, provided with sterilized bedding and fed *ad libitum* with a commercial diet sterilized by gamma irradiation (4 Mrad) and autoclaved drinking water. The germ-free status was confirmed by testing fecal samples for aerobic and anaerobic bacteria and yeast.

#### Experimental design

As usually done for axenic mice, batches of three to four mice were used per experiment. On day 0, each mouse was inoculated individually and intragastrically with 10^8^ CFU of *E*. *coli* MG/pZE1, MG/intI1 or MG/intI1* in 500 μl BHI. In parallel, 100 μl of each bacterial culture were plated on LB agar supplemented with tobramycin (Tobra, 100 μg/ml) to control the absence of tobramycin-resistant recombinant within the inoculum. Fecal pellets from each mouse were collected individually in sterile tubes after spontaneous emission, at least three days a week. For each mouse, one fresh pellet was weighed and used directly for CFU counting and the remaining pellets were kept at -80°C for further analysis. For the experiment with ciprofloxacin, the antibiotic was added directly into the drinking water of mice on day 11 post inoculation, just after the sampling of fecal pellets on that day. The water bottles were changed every 72h. Ciprofloxacin concentration in mice faeces was measured as described in S1 Materials and Methods.

### Bacterial counting and plasmid stability in mice gut

Fecal pellets were resuspended at 10 mg/ml in sterile 1X phosphate buffer saline (PBS), and 10-fold dilutions in 1X PBS were plated on non-selective LB agar medium to monitor the intestinal bacterial colonization, and on selective LB agar media supplemented with the appropriate antibiotics to monitor the stability of plasmids pZE1, pZE1intI1 and pZE1intI1* (Amp, 100 μg/ml) and p6851 (kanamycin (Km), 25 μg/ml). CFU were counted using the Wasp 2 spiral system (AES Chemunex, Bruz, France). The detection limit was 100 CFU/g of faeces. For each experiment, results were expressed as the average CFU/g of faeces ± standard error (SD) for all mice (data-sheets A, H and J in S1 Data).

### Ciprofloxacin susceptibility testing

The minimal inhibitory concentration of ciprofloxacin was determined with E-test strips following the supplier’s instructions (BioMérieux, France).

### Recombination activity assay

The activity of the IntI1_R32_H39_ integrase was determined as previously described [24] (S1 Fig). Briefly, for *in vitro* recombination activity assay, planktonic cultures of MG/intI1 and MG/intI1* were grown in LB for 24 hours with shaking. Ten-fold dilutions of cultures were plated on LB agar + Km + Amp and LB agar + Tobra (10 μg/ml), to estimate CFU/ml. For *in vivo* recombination activity assay, fecal pellets collected from mice colonized with MG/intI1 and MG/intI1* were weighed and resuspended at 10 mg/ml in sterile 1X PBS. Ten-fold dilutions of samples were plated on LB agar + Km + Amp and LB agar + Tobra to estimate CFU/g of faeces (data-sheets B and F in S1 Data). The frequency of recombinants (FR) was calculated as the ratio of the CFU/ml or CFU/g of faeces on LB agar + Tobra to the CFU/ml or CFU/g of faeces on LB agar + Amp + Km for *in vitro* and *in vivo* assays, respectively. All assays were performed at least five times in triplicate for 24-h-old planktonic cultures (data-sheet C in S1 Data), and with one fecal pellet from each mouse on each sampling day (average of three to four mice each day depending on the experiment; data-sheets B and F in S1 Data). Detection limit was 100 CFU/g of faeces in the mouse model. Deletion of the synthetic cassette *cat(T4)-attC*_VCR2_ was verified by PCR and Sanger sequencing using primers aac(6’)-Ib-R and MRV-D2 primers (S2 Table).

### Competition assays

#### Competition experiment

The recombined p6851 plasmid was first purified from a recombinant clone (resistant to tobramycin and susceptible to chloramphenicol (Tobra^R^Cm^S^)) recovered from faeces of mice colonized with MG/intI1 strain and treated with ciprofloxacin. The proper recombination was verified by PCR and Sanger sequencing, and the plasmid was transformed in strain MG1656*λatt*::*gfp* (strain expressing constitutively the green fluorescent protein GFP; Table 1). The fitness of the MG1656 strain carrying the native non-recombined p6851 plasmid, relative to the MG1656*λatt*::*gfp* carrying the recombined p6851 plasmid, was estimated in pairwise competition assays as previously described [36]. Briefly, independent pre-cultures of both competitors were grown in DM25 medium overnight and mixed at a 1:1 volume ratio the following day. Fresh DM25 cultures were then inoculated with this mix performing a 100-fold dilution. Cultures were then propagated for 4 days with a daily 100-fold dilution in fresh medium. Samples were collected each day to follow the relative frequency of the two competitors by flow cytometry.

#### Flow cytometry analysis

The relative frequency of bacteria expressing GFP was determined with a Beckman Coulter CytoFLEX LX flow cytometer (Beckman Coulter, Brea, CA, USA). Measurements were performed directly on bacterial cultures. Bacterial cells were first gated on the basis of their distribution on forward (FSC-A) *versus* side scatter (SSC-A) plots. Only single-cell bacteria were counted using a FSC-W *versus* SSC-A plot. GFP single cells were then separated from non-GFP single cells by using the fluorescent marker. Green fluorescence was acquired on the B525-FITC-A channel (525 ± 15 nm). A fluorescence threshold was determined based on the fluorescence distribution of a GFP-positive control and GFP- negative control; this threshold remained constant throughout all the experiments. The relative frequency of the two competitors was calculated by counting 50 000 single cells. All measurements were made in triplicate. Controls were performed to assess the level of false positives and false negatives in GFP quantification. Both were shown to be negligible and constant throughout independent daily measurements. For each competition assay, the selection coefficient of the non-GFP strain was determined from the slope of the regression ln [freq_nonGFP_/freq_GFP_] plotted against the time course in generations [69]. Experiments were performed twice with 6 replicates per experiment (data-sheet I in S1 Data). The fluorescent marker used in the MG1656*λatt*::*gfp* strain had no cost when compared with the parental MG1656 strain [36].

### Quantitative Real-Time PCR

Total RNA was isolated from fecal pellets by using the PowerMicrobiome RNA Isolation Kit (Mo Bio Laboratories, Inc., Carlsbad, CA, USA) and from 24-h-old-planktonic cultures using the NucleoSpinRNA kit (Macherey-Nagel, Düren, Germany), according to the manufacturers’ instructions. RNA samples were treated with DNase (Turbo DNase; Ambion, Austin, TX, USA). The quality, integrity and concentration of total prokaryotic RNA were evaluated with an Agilent 2100 Bioanalyzer (Agilent, Böblingen, Germany). cDNA was synthesized with the PrimeScript RT reagent kit (Perfect Real Time) (Takara, Otsu, Japan) according to the manufacturer’s instructions. qRT-PCR were performed on an Mx3005P apparatus (Stratagene-Agilent Technologies, Inc., Santa Clara, CA, USA) in triplicate in a 25μl reaction mix with PerfectaqPCR SuperMix (Quanta Biosciences, Gaithersburg, MD, USA), following the supplier’s instructions, and with specific primers and Taqman probes for *dinD*, *recN*, *sfiA*, *intI1* genes and the housekeeping gene *dxs* (S2 Table). The PCR cycling conditions consisted of one cycle at 95°C for 10 min, and 40 cycles at 95°C for 30 sec and 60°C for 1 min. The absolute quantification of transcripts for *dinD*, *recN*, *sfiA* and *intI1* genes was normalized to the absolute quantification of the housekeeping gene *dxs* for each sample (at least three 24-h-old-planktonic cultures or three mice per condition; data-sheets D, E and G in S1 Data). *dxs* is a single-copy chromosomal *E*. *coli* gene encoding for D-1-deoxyxylulose 5-phosphate synthase and it has been previously used for normalization [38,70].

### Verification of the integrase gene integrity after ciprofloxacin treatment

Ten recombinant clones resistant to tobramycin and susceptible to chloramphenicol were recovered from faeces of mice colonized with MG/intI1 strain and treated with ciprofloxacin. Plasmid extractions were performed using the NucleoSpin mini kit (Macherey Nagel) and pZE1intI1 plasmid was further isolated. The region encompassing the PintI1 promoter to the end of *intI1* gene was first amplified by PCR using primers ApXFP3 and pZE12rev (S2 Table) with the PrimeSTAR Max DNA Polymerase (Takara) following the manufacturer’s instructions. PCR products were analysed by Sanger sequencing with primers ApXFP3, Int1LC18 and pZE12rev (S2 Table).

### Statistical analysis

Differences in bacterial colonization and plasmid stability in the gut were determined using the Student’s paired *t* test. Differences in recombination frequencies and quantification of transcript levels were determined using the Mann-Whitney *U* test (comparing two groups).

## Supporting information

S1 Fig Recombination activity assay. **(A)** Description of pZE1 derivative plasmids: plasmid pZE1 (control plasmid that did not carry the *intI1* gene), plasmid pZE1intI1 (expression of *intI1* from the LexA-regulated (SOS-regulated) wild-type integrase promoter P*intI1*), and plasmid pZE1intI1* (constitutive expression of *intI1* from the derepressed P*intI1* carrying mutations in the LexA-binding site (indicated by a black star) inhibiting LexA binding); **(B)** Description of p6851 plasmid before (native p6851) and after recombination (Recombined p6851). Native p6851 carries a constitutively expressed *cat(T4)* gene located between two *attC* sites (*attC*_*aadA7*_ and *attC*_*VCR2*_), a P*lacZ* promoter upstream of *attC*_*aadA7*_ and the non-expressed aac(6’)-Ib* gene downstream of *attC*_*VCR2*_. Recombined p6851 has lost the *attC*_*aadA7-*_*cat(T4)*-*attC*_*VCR2*_ structure and allows the constitutive expression of the aac(6’)-Ib* gene from P*lacZ*. For each plasmid, the resistance phenotype is indicated below in bold: *bla*, *aphA3*, *cat(T4)* and aac(6’)-Ib* genes confer resistance to ampicillin (Amp^R^), kanamycin (Km^R^), chloramphenicol (Cm^R^) and tobramycin (Tobra^R^) respectively. The integrase activity assay was performed as previously described [24]. *E*. *coli* MG1656 cells were freshly electroporated simultaneously with native p6851 plasmid and one of the pZE1-derivative. The assay is based on the IntI1_R32_H39_ integron integrase capability to excise the synthetic cassette *cat(T4)*-*attC*_*VCR2*_ (carried on native p6851) by catalysing the specific recombination between the *attC*_*aadA7*_ and *attC*_*VCR2*_ sites, allowing functional aminoglycoside acetyltransferase-6’ synthesis that confers selectable resistance to tobramycin (recombined p6851; expression of aac(6’)-Ib* from P*lacZ* promoter). Integrase activity was determined as the frequency of recombinants calculated as the ratio of the CFU/ml or CFU/g of faeces (respectively for *in vitro* and *in vivo* assays) on LB + Tobra to the CFU/ml or CFU/g of faeces on LB + Amp + Km.
(PDF)

S2 Fig Emergence of tobramycin-resistant recombinants in the mouse gut. On day 0, two groups of germ-free mice were inoculated with respectively 10^8^ CFU of MG/intI1 (carrying p6851 and pZE1intI1 allowing the expression of *intI1* SOS-regulated, n = 4) **(A)** or MG/intI1* (carrying p6851 and pZE1intI1* allowing the constitutive expression of *intI1*, n = 3) **(B)**. Emergence of tobramycin-resistant recombinants in the mouse gut was monitored by counting the CFU/g of faeces on selective medium supplemented with tobramycin. Total bacterial population carrying the two plasmids was monitored by counting the CFU/g of faeces on selective medium supplemented with kanamycin (p6851) and ampicillin (pZE1intI1 and pZE1intI1*). Symbols represent the average of the CFU/g of faeces per day and error bars indicate the SD.
(PDF)

S3 Fig Fitness cost of native p6851 versus recombined p6851. Strain MG1656*λatt*::*gfp* carrying the recombined p6851 plasmid was competed against strain MG1656 carrying the native non-recombined p6851 in pairwise competition assays. The median selection coefficient of the MG1656/native p6851 is represented by a black line (s = 0.002) and was not statistically different from zero (p = 0.62 using one Sample Wilcoxon signed rank test,) indicating that the two strains had similar fitness. Data represent results of 12 independent competition assays.
(PDF)

S4 Fig Effect of ciprofloxacin on bacterial survival, plasmid stability and emergence of tobramycin-resistant recombinants in the mouse gut. On day 0, three germ-free mice were inoculated with 10^8^ CFU of MG/intI1 (carrying p6851 and pZE1intI1 allowing the expression of *intI1* SOS-regulated). Ciprofloxacin was added to the drinking water of mice on day 11 post inoculation just after fecal sampling on that day. **(A)** Bacterial colonization in the mouse gut was monitored by counting the CFU/g of faeces on non-selective medium (MG1656). Plasmid stability was evaluated by counting the CFU/g of faeces on selective media supplemented with kanamycin (p6851) or ampicillin (pZE1intI1). **(B)** Emergence of tobramycin-resistant recombinants in the mouse gut was monitored by counting the CFU/g of faeces on selective medium supplemented with tobramycin. Total bacterial population carrying the two plasmids was monitored by counting the CFU/g of faeces on selective medium supplemented with kanamycin and ampicillin. Symbols represent the average of the CFU/g of faeces per day and error bars indicate the SD. Differences were determined using the Student’s paired *t* test. *p < 0.05.
(PDF)

S1 Table Ciprofloxacin concentration in mice faeces and its effect on bacterial survival in the gut. (DOCX)

S2 Table Primers and probes used in this study. (DOCX)

S1 Materials and Methods Measurement of ciprofloxacin concentration in mice faeces. (DOCX)

S1 Data Raw data of bacterial counts, transcripts levels estimation, frequency of recombinants estimation and fitness cost. (XLSX)
