# Supplementary material for: Activation of class 1 integron integrase is promoted in the intestinal environment
Source: PLoS Genet. 2022 Apr 28;18(4):e1010177. doi: 10.1371/journal.pgen.1010177 (PMC9090394; doi:10.1371/journal.pgen.1010177)
Supplement: S1 Fig — (A) Description of pZE1 derivative plasmids: plasmid pZE1 (control plasmid that did not carry the intI1 gene), plasmid pZE1intI1 (expression of intI1 from the LexA-regulated (SOS-regulated) wild-type integrase promoter PintI1), and plasmid pZE1intI1* (constitutive expression of intI1 from the derepressed PintI1 carrying mutations in the LexA-binding site (indicated by a black star) inhibiting LexA binding); (B) Description of p6851 plasmid before (native p6851) and after recombination (Recombined p6851). Native p6851 carries a constitutively expressed cat(T4) gene located between two attC sites (attCaadA7 and attCVCR2), a PlacZ promoter upstream of attCaadA7 and the non-expressed aac(6’)-Ib* gene downstream of attCVCR2. Recombined p6851 has lost the attCaadA7-cat(T4)-attCVCR2 structure and allows the constitutive expression of the aac(6’)-Ib* gene from PlacZ. For each plasmid, the resistance phenotype is indicated below in bold: bla, aphA3, cat(T4) and aac(6’)-Ib* genes confer resistance to ampicillin (AmpR), kanamycin (KmR), chloramphenicol (CmR) and tobramycin (TobraR) respectively. The integrase activity assay was performed as previously described [24]. E. coli MG1656 cells were freshly electroporated simultaneously with native p6851 plasmid and one of the pZE1-derivative. The assay is based on the IntI1R32_H39 integron integrase capability to excise the synthetic cassette cat(T4)-attCVCR2 (carried on native p6851) by catalysing the specific recombination between the attCaadA7 and attCVCR2 sites, allowing functional aminoglycoside acetyltransferase-6’ synthesis that confers selectable resistance to tobramycin (recombined p6851; expression of aac(6’)-Ib* from PlacZ promoter). Integrase activity was determined as the frequency of recombinants calculated as the ratio of the CFU/ml or CFU/g of faeces (respectively for in vitro and in vivo assays) on LB + Tobra to the CFU/ml or CFU/g of faeces on LB + Amp + Km. (PDF) [file pgen.1010177.s001.pdf]

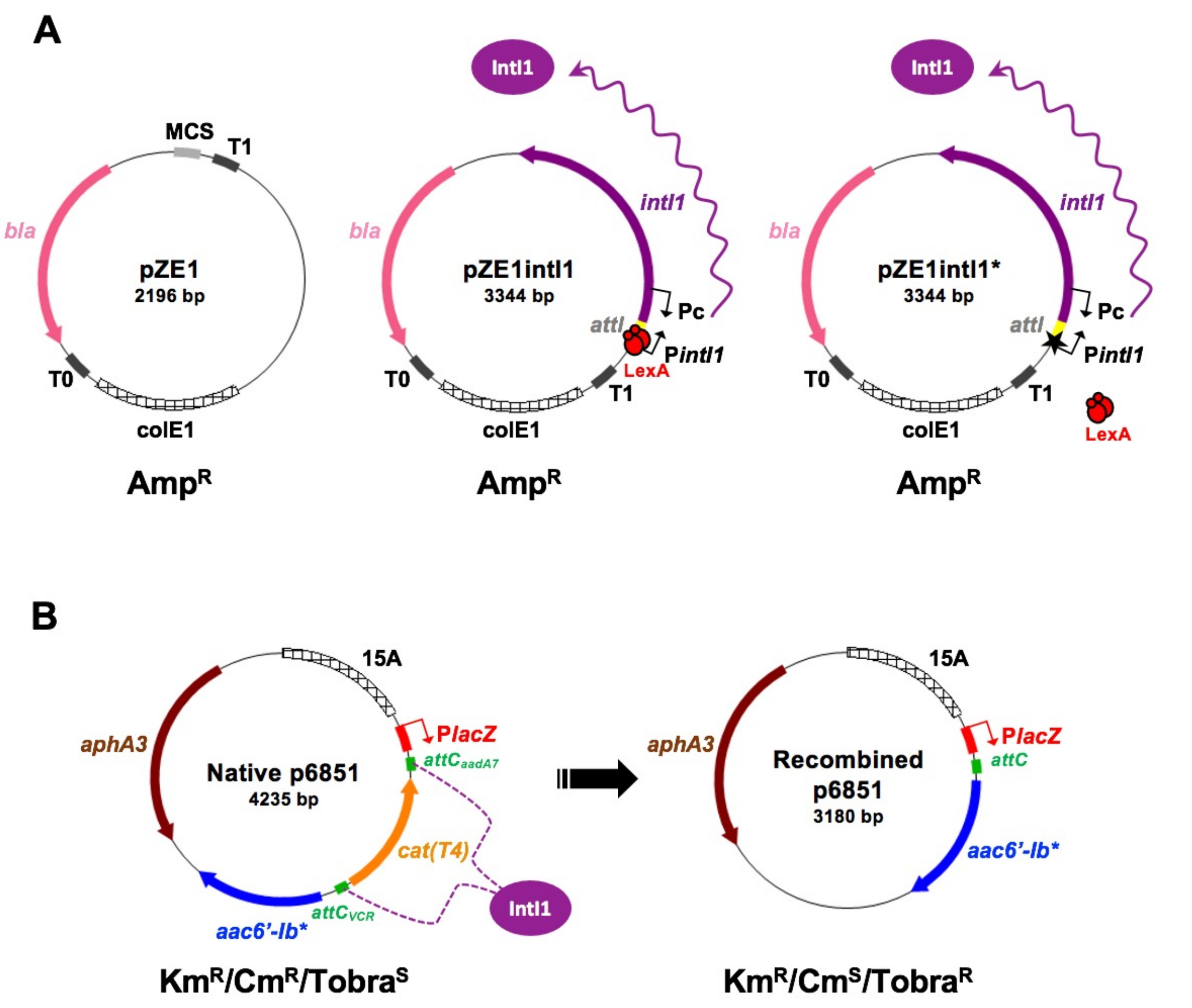

S1 Fig. Recombination activity assay

(A) Description of pZE1 derivative plasmids : plasmid pZE1 (control plasmid that did not carry the *intI1* gene), plasmid pZE1intI1 (expression of *intI1* from the LexA-regulated (SOS-regulated) wild-type integrase promoter P*intI1*), and plasmid pZE1intI1* (constitutive expression of *intI1* from the derepressed P*intI1* carrying mutations in the LexA-binding site (indicated by a black star) inhibiting LexA binding); **(B)** Description of p6851 plasmid before (native p6851) and after recombination (Recombined p6851). Native p6851 carries a constitutively expressed *cat(T4)* gene located between two *attC* sites ($attC_{aadA7}$ and $attC_{VCR2}$), a P*lacZ* promoter upstream of $attC_{aadA7}$ and, and the non-expressed *aac(6')-Ib** gene downstream of $attC_{VCR2}$. Recombined p6851 has lost the $attC_{aadA7}$-*cat(T4)*-$attC_{VCR2}$ structure and allows the constitutive expression of the *aac(6')-Ib** gene from P*lacZ*. For each plasmid, the resistance phenotype is indicated below in bold: *bla*, *aphA3*, *cat(T4)* and *aac(6')-Ib** genes confer resistance to ampicillin (Amp^R), kanamycin (Km^R), chloramphenicol (Cm^R) and tobramycin ($Tobra^R$) respectively.

The integrase activity assay was performed as previously described [24]. *E. coli* MG1656 cells were freshly electroporated simultaneously with native p6851 plasmid and one of the pZE1-derivative. The assay is based on the $IntI1_{R32\_H39}$ integron integrase capability to excise the synthetic cassette *cat(T4)*-$attC_{VCR2}$ (carried on native p6851) by catalysing the specific recombination between the $attC_{aadA7}$ and $attC_{VCR2}$ sites, allowing functional aminoglycoside acetyltransferase-6' synthesis that confers selectable resistance to tobramycin (recombined p6851; expression of *aac(6')-Ib** from P*lacZ* promoter). Integrase activity was determined as the frequency of recombinants calculated as the ratio of the CFU/ml or CFU/g of faeces (respectively for *in vitro* and *in vivo* assays) on LB + Tobra to the CFU/ml or CFU/g of faeces on LB + Amp + Km.
