# Supplementary material for: Activation of class 1 integron integrase is promoted in the intestinal environment
Source: PLoS Genet. 2022 Apr 28;18(4):e1010177. doi: 10.1371/journal.pgen.1010177 (PMC9090394; doi:10.1371/journal.pgen.1010177)
Supplement: S2 Fig — On day 0, two groups of germ-free mice were inoculated with respectively 108 CFU of MG/intI1 (carrying p6851 and pZE1intI1 allowing the expression of intI1 SOS-regulated, n = 4) (A) or MG/intI1* (carrying p6851 and pZE1intI1* allowing the constitutive expression of intI1, n = 3) (B). Emergence of tobramycin-resistant recombinants in the mouse gut was monitored by counting the CFU/g of faeces on selective medium supplemented with tobramycin. Total bacterial population carrying the two plasmids was monitored by counting the CFU/g of faeces on selective medium supplemented with kanamycin (p6851) and ampicillin (pZE1intI1 and pZE1intI1*). Symbols represent the average of the CFU/g of faeces per day and error bars indicate the SD. (PDF) [file pgen.1010177.s002.pdf]

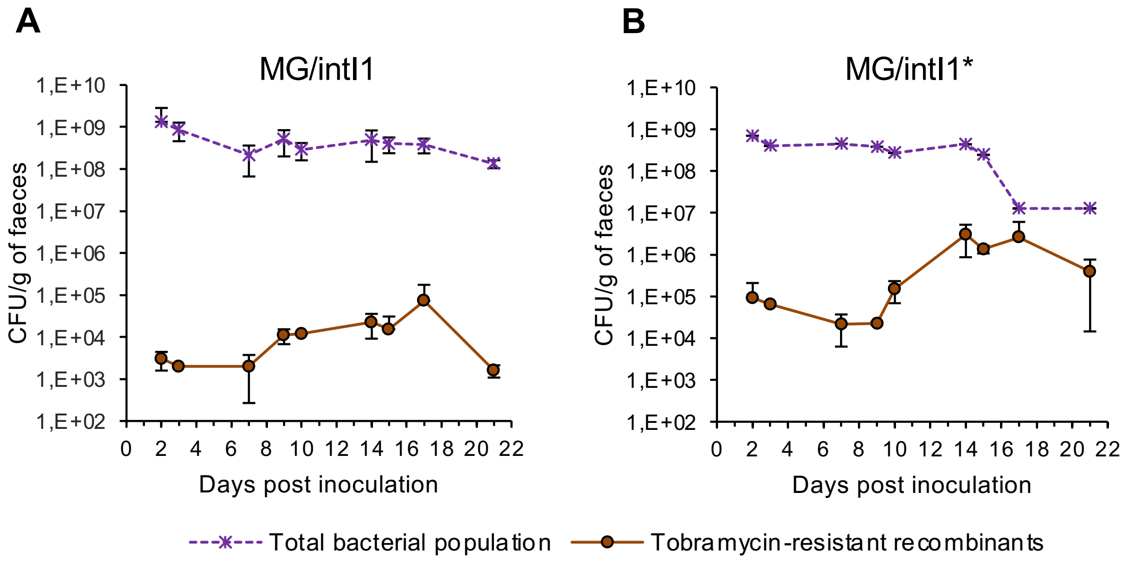

S2 Fig. Emergence of tobramycin-resistant recombinants in the mouse gut.

On day 0, two groups of germ-free mice were inoculated with respectively 10^8 CFU of MG/intI1 (carrying p6851 and pZE1intI1 allowing the expression of *intI1* SOS-regulated, n = 4) **(A)** or MG/intI1* (carrying p6851 and pZE1intI1* allowing the constitutive expression of *intI1*, n = 3) **(B)**. Emergence of tobramycin-resistant recombinants in the mouse gut was monitored by counting the CFU/g of faeces on selective medium supplemented with tobramycin. Total bacterial population carrying the two plasmids was monitored by counting the CFU/g of faeces on selective medium supplemented with kanamycin (p6851) and ampicillin (pZE1intI1 and pZE1intI1*). Symbols represent the average of the CFU/g of faeces per day and error bars indicate the SD.
