# Supplementary material for: Activation of class 1 integron integrase is promoted in the intestinal environment
Source: PLoS Genet. 2022 Apr 28;18(4):e1010177. doi: 10.1371/journal.pgen.1010177 (PMC9090394; doi:10.1371/journal.pgen.1010177)
Supplement: S3 Fig — Strain MG1656λatt::gfp carrying the recombined p6851 plasmid was competed against strain MG1656 carrying the native non-recombined p6851 in pairwise competition assays. The median selection coefficient of the MG1656/native p6851 is represented by a black line (s = 0.002) and was not statistically different from zero (p = 0.62 using one Sample Wilcoxon signed rank test,) indicating that the two strains had similar fitness. Data represent results of 12 independent competition assays. (PDF) [file pgen.1010177.s003.pdf]

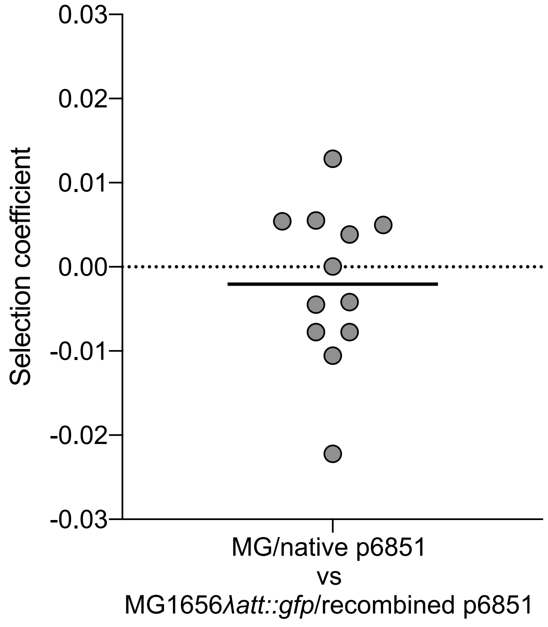

S3 Fig. Fitness cost of native p6851 versus recombined p6851

Strain MG1656*λatt::gfp* carrying the recombined p6851 plasmid was competed against strain MG1656 carrying the native non-recombined p6851 in pairwise competition assays. The median selection coefficient of the MG1656/native p6851 is represented by a black line (s = 0.002) and was not statistically different from zero (p = 0.62 using one Sample Wilcoxon signed rank test,) indicating that the two strains had similar fitness. Data represent results of 12 independent competition assays.
