# Supplementary material for: Activation of class 1 integron integrase is promoted in the intestinal environment
Source: PLoS Genet. 2022 Apr 28;18(4):e1010177. doi: 10.1371/journal.pgen.1010177 (PMC9090394; doi:10.1371/journal.pgen.1010177)
Supplement: S4 Fig — On day 0, three germ-free mice were inoculated with 108 CFU of MG/intI1 (carrying p6851 and pZE1intI1 allowing the expression of intI1 SOS-regulated). Ciprofloxacin was added to the drinking water of mice on day 11 post inoculation just after fecal sampling on that day. (A) Bacterial colonization in the mouse gut was monitored by counting the CFU/g of faeces on non-selective medium (MG1656). Plasmid stability was evaluated by counting the CFU/g of faeces on selective media supplemented with kanamycin (p6851) or ampicillin (pZE1intI1). (B) Emergence of tobramycin-resistant recombinants in the mouse gut was monitored by counting the CFU/g of faeces on selective medium supplemented with tobramycin. Total bacterial population carrying the two plasmids was monitored by counting the CFU/g of faeces on selective medium supplemented with kanamycin and ampicillin. Symbols represent the average of the CFU/g of faeces per day and error bars indicate the SD. Differences were determined using the Student’s paired t test. *p < 0.05. (PDF) [file pgen.1010177.s004.pdf]

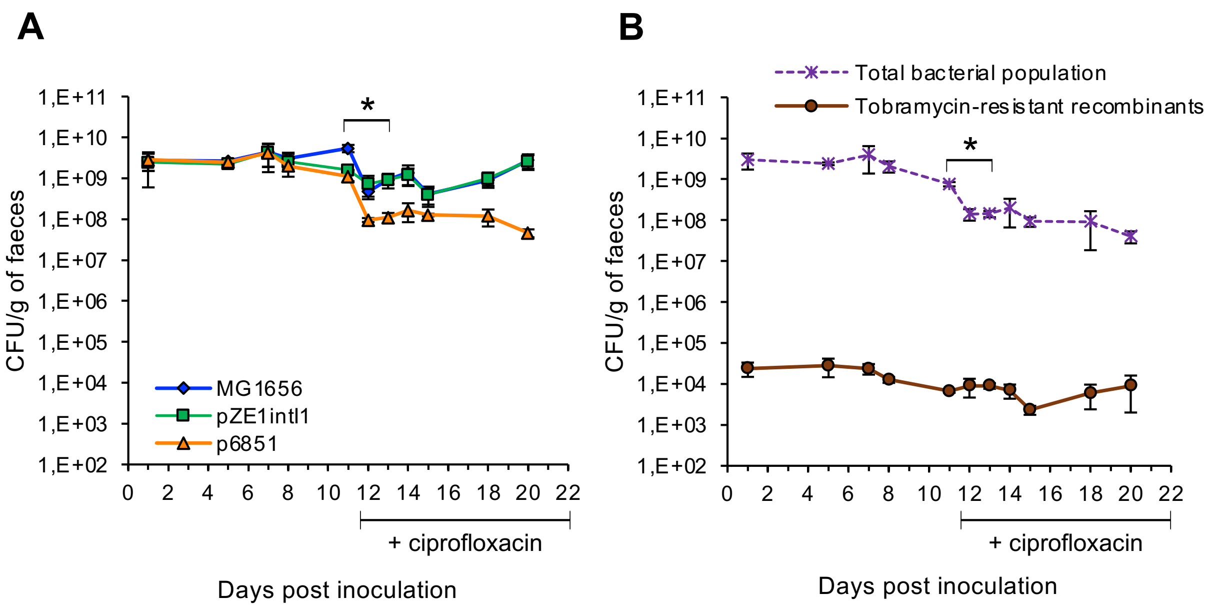

S4 Fig. Effect of ciprofloxacin on bacterial survival, plasmid stability and emergence of tobramycin-resistant recombinants in the mouse gut.

On day 0, three germ-free mice were inoculated with 10^8 CFU of MG/intI1 (carrying p6851 and pZE1intI1 allowing the expression of *intI1* SOS-regulated). Ciprofloxacin was added to the drinking water of mice on day 11 post inoculation just after fecal sampling on that day. **(A)** Bacterial colonization in the mouse gut was monitored by counting the CFU/g of faeces on non-selective medium (MG1656). Plasmid stability was evaluated by counting the CFU/g of faeces on selective media supplemented with kanamycin (p6851) or ampicillin (pZE1intI1). **(B)** Emergence of tobramycin-resistant recombinants in the mouse gut was monitored by counting the CFU/g of faeces on selective medium supplemented with tobramycin. Total bacterial population carrying the two plasmids was monitored by counting the CFU/g of faeces on selective medium supplemented with kanamycin and ampicillin. Symbols represent the average of the CFU/g of faeces per day and error bars indicate the SD. Differences were determined using the Student's paired *t* test. *$p < 0.05$.
