# Supplementary material for: Activation of class 1 integron integrase is promoted in the intestinal environment
Source: PLoS Genet. 2022 Apr 28;18(4):e1010177. doi: 10.1371/journal.pgen.1010177 (PMC9090394; doi:10.1371/journal.pgen.1010177)
Supplement: S1 Table — (DOCX) [file pgen.1010177.s005.docx]

**S1 Table. Ciprofloxacin concentration in mice faeces and its effect on bacterial survival in the gut.**

| **Ciprofloxacin concentration in the drinking water (µg/ml)** | **Ciprofloxacin dose (mg/kg of body weight/day) ^a^** | **Ciprofloxacin concentration in mice ^b^ faeces**  **(µg/g of faeces)** | **Total bacterial population ^c^**  **(CFU/g of faeces)** | |
| --- | --- | --- | --- | --- |
|  |  |  | **Before**  **ciprofloxacin** | **After 3 days**  **of ciprofloxacin** |
| 0.4 | 0.1 | 0.3 ± 0.2**^d^** | 5.4 ± 1.1 x 10^9^ **^d^** | 9.2 ± 5.6 x 10^8^**^d^** |
| 4 | 1 | 1.3 ± 0.1**^e^** | 5.6 ± 1.6 x 10^9^**^e^** | < 10^2^ |
| 40 | 10 | 18.2 ± 6.8**^e^** | 5.3 ± 0.2 x 10^9^ **^e^** | < 10^2^ |

**^a^** The dose was calculated from the ciprofloxacin concentration in the drinking water assuming that C3H mice weighing 20 g drink around 5 ml per day (1).

**^b^** Gnotobiotic mice colonized with *E. coli* MG/intI1.

**^c^** Total bacterial population counted on non-selective Lysogeny Broth agar medium.

**^d^** Mean ± SD (n=3).

**^e^** Mean ± SD (n=2).

Reference:

1 Bachmanov AA, Reed DR, Beauchamp GK, Tordoff MG. 2002. Food intake, water intake, and drinking spout side preference of 28 mouse strains. Behav Genet 32:435-43.
