# Supplementary material for: Activation of class 1 integron integrase is promoted in the intestinal environment
Source: PLoS Genet. 2022 Apr 28;18(4):e1010177. doi: 10.1371/journal.pgen.1010177 (PMC9090394; doi:10.1371/journal.pgen.1010177)
Supplement: S2 Table — (DOCX) [file pgen.1010177.s006.docx]

**S2 Table. Primers and probes used in this study.**

| **Name** | **Sequence (5’ to 3’)** | **Reference** |
| --- | --- | --- |
| **Primers** |  |  |
| aac6’-Ib-R | CATAGAGCATCGCAAGGTCA | This work |
| MRV-D2 | TTCTGCTGACGCACCGGTG | This work |
| dxs-LC3 | ATGACGTGGCGATTCAAAA | This work |
| dxs-LC4 | AGCCGGTATAGAGCATCTGG | This work |
| intI1-LC1 | GCCTTGATGTTACCCGAGAG | (1) |
| intI1-LC5 | GATCGGTCGAATGCGTGT | (1) |
| sfiA-L1 | TTACAGCAACTCGGTCAGCA | This work |
| sfiA-R1 | CAGTGTGGCAAGGGGAGA | This work |
| dinD-L3 | AATTGGTTGAGGCAGCACAG | This work |
| dinD-R3 | AGATTAGCCGCCAGTTCTGT | This work |
| recN-L3 | AGCTACCATCCAGATTGCTGA | This work |
| recN-R2 | CGACTGGTAATACTGTGGCA | This work |
| **Probes** |  |  |
| dxs-probe | [6FAM]ATTATGACCCCGAGCGATGAAAACGAA[TAM] | This work |
| intI1-probe | [6FAM]ATTCCTGGCCGTGGTTCTGGGTTTT[BHQ1] | (1) |
| sfiA-probe | [6FAM]CTGGGCTACCCTTAACGAAAGTAATGCAGA[TAM] | This work |
| dinD-probe | [6FAM]CCAGGGGCTGTATGGTGGATTAGATCAGA[TAM] | This work |
| recN-probe | [6FAM]CCCAACCGACTATTTGAACTTGAACAGCGC[TAM] | This work |

Reference:

1 Barraud O, Baclet MC, Denis F, Ploy MC. 2010. Quantitative multiplex real-time PCR for detecting class 1, 2 and 3 integrons. *J Antimicrob Chemother* **65**:1642-1645.
