# Supplementary material for: Activation of class 1 integron integrase is promoted in the intestinal environment
Source: PLoS Genet. 2022 Apr 28;18(4):e1010177. doi: 10.1371/journal.pgen.1010177 (PMC9090394; doi:10.1371/journal.pgen.1010177)
Supplement: S1 Materials and Methods — (DOCX) [file pgen.1010177.s007.docx]

**S1 Materials and Methods**

**Measurement of ciprofloxacin concentration in mice faeces.** Ciprofloxacin concentrations were measured by LC-MS/MS as previously described with slight modifications (1,2). 1X PBS was added to faeces (10/1 ratio) and the mixture was sonicated, vortexed and centrifuged. The supernatant was extracted with dichloromethane. The organic phase was transferred into a clean tube and evaporated at 40°C under N2 flow. One hundred and fifty microliters of mobile phase diluted in 10 mM ammonium formiate, were added to the dry residue and mixed for 5 min. The mixture was centrifuged at 1000 g for 5 min and 15 µl of the supernatant were injected into an API3000 LC-MS/MS system (Foster City, CA, USA). Separation was achieved using a XBridge BEH300 C18 Peptide Separation Technology (PST) column (Waters Corporation, Milford, MA, USA) and a mobile phase of acetonitrile/H_2_O/formic (25/75/0.1) with flow rate of 0.2 ml/min. Retention times were approximately 2 min for ciprofloxacin and 2.2 min for moxifloxacin used as an internal standard. Electrospray ionization (ESI) mass spectrometry with multiplereaction monitoring (MRM) of positive ions was used for detection. The ions monitored were 332.1→314.10 for ciprofloxacin and 402.1→384.0 for moxifloxacin. The ratio of analyte product ion peak area to that of the internal standard was used for quantitation. The intra-day accuracy of the ciprofloxacin standards did not exceed 7%. The intra-day precision for the determination of ciprofloxacin was within ± 8% and the intra-day precision for the determination of ciprofloxacin quality controls was between ± 11%. The limit of quantification was estimated at 0.03 µg/g of faeces.

References:

1. Gontijo AV, Brillault J, Gregoire N, Lamarche I, Gobin P, Couet W, et al. Biopharmaceutical characterization of nebulized antimicrobial agents in rats: 1. Ciprofloxacin, moxifloxacin, and grepafloxacin. Antimicrob Agents Chemother. juill 2014;58(7):3942‑9.

2. Gregoire N, Raherison S, Grignon C, Comets E, Marliat M, Ploy MC, et al. Semimechanistic pharmacokinetic-pharmacodynamic model with adaptation development for time-kill experiments of ciprofloxacin against Pseudomonas aeruginosa. Antimicrob Agents Chemother. juin 2010;54(6):2379‑84.
